# Segmentation of mediastinal lymph nodes in CT with anatomical priors

**DOI:** 10.1007/s11548-024-03165-4

**Published:** 2024-05-13

**Authors:** Tejas Sudharshan Mathai, Bohan Liu, Ronald M. Summers

**Affiliations:** 1 Clinical Center, National Institutes of Health (NIH), Bethesda, MD USA; 2 Department of Radiology, School of Medicine and Health Sciences, George Washington University, Washington, DC USA

**Keywords:** CT, Lymph node, Mediastinum, Segmentation, Anatomical priors, Deep learning

## Abstract

**Purpose:**

Lymph nodes (LNs) in the chest have a tendency to enlarge due to various pathologies, such as lung cancer or pneumonia. Clinicians routinely measure nodal size to monitor disease progression, confirm metastatic cancer, and assess treatment response. However, variations in their shapes and appearances make it cumbersome to identify LNs, which reside outside of most organs.

**Methods:**

We propose to segment LNs in the mediastinum by leveraging the anatomical priors of 28 different structures (e.g., lung, trachea etc.) generated by the public TotalSegmentator tool. The CT volumes from 89 patients available in the public NIH CT Lymph Node dataset were used to train three 3D off-the-shelf nnUNet models to segment LNs. The public St. Olavs dataset containing 15 patients (out-of-training-distribution) was used to evaluate the segmentation performance.

**Results:**

For LNs with short axis diameter $\ge$ 8 mm, the 3D cascade nnUNet model obtained the highest Dice score of 67.9 ± 23.4 and lowest Hausdorff distance error of 22.8 ± 20.2. For LNs of all sizes, the Dice score was 58.7 ± 21.3 and this represented a $\ge$10% improvement over a recently published approach evaluated on the same test dataset.

**Conclusion:**

To our knowledge, we are the first to harness 28 distinct anatomical priors to segment mediastinal LNs, and our work can be extended to other nodal zones in the body. The proposed method has the potential for improved patient outcomes through the identification of enlarged nodes in initial staging CT scans.

**Supplementary Information:**

The online version contains supplementary material available at 10.1007/s11548-024-03165-4.

## Introduction

Lymph nodes and the lymphatic system comprise an integral part of the body’s natural defense mechanisms and play a vital role in maintaining a person’s health. Abnormalities to the lymphatic system can result in enlarged lymph nodes (lymphadenopathy) [1, 2] with etiologies ranging from infection, autoimmune disease or malignancy. Distinguishing between the causes for enlarged and metastatic nodes from non-metastatic LNs is critical for clinicians in determining the correct treatment [1–4]. Frequently, radiologists use a systematic approach to identify suspicious nodes through nodal size measurement with the help of established guidelines, such as the tumor, node, and metastasis (TNM) criteria [4]. In particular, the presence of enlarged LNs in the setting of cancer not only dictates the staging and extent, but is vital to treatment and management.

In clinical practice, radiologists routinely identify, manually measure, and describe the features of lymph nodes on CT and MRI to identify areas of pathology. Among the various imaging features for lymphadenopathy, nodal size is the most widely used criteria [1–4] to determine benign versus malignant status when paired with clinical data. A node is considered enlarged if its short-axis diameter (SAD) is greater than 10 mm on an axial CT slice [1–5]. However, this assessment can be cumbersome and time-consuming, especially at initial staging and while comparing multiple sites of metastasis during the evaluation of treatment response in follow-up imaging. To help relieve this laborious process, automated LN measurement can augment radiology workflows by aiding in the identification of LNs in specific regions of the body, such as the mediastinum.

**Fig. 1:**
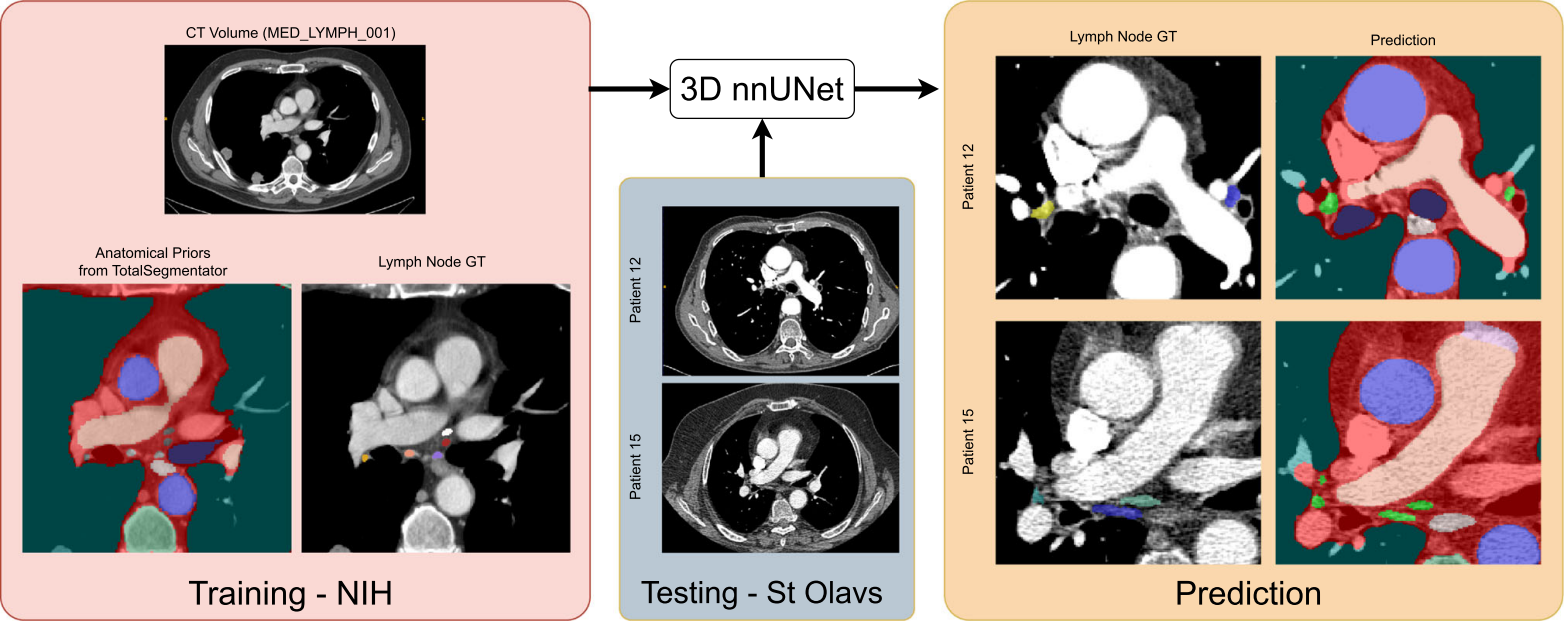
Flowchart of the proposed approach to segment mediastinal lymph nodes in CT using anatomical priors. First, the public TotalSegmentator tool was used to segment 28 structures in 89 mediastinal CT volumes from the public NIH CT Lymph Node dataset. Next, these labels were combined with the manual annotations for mediastinal LNs, and used to train a 3D nnUNet segmentation model. At test time, the 3D nnUNet was executed on CT volumes of 15 patients in the public St Olavs dataset. Green labels in the prediction correspond to the predicted LNs. The figure is best viewed in color in the PDF

Several approaches have been proposed to detect and segment mediastinal lymph nodes in both CT [6–13] and MRI [14–20]. Only a handful [6, 8, 9, 11–13] exploit the anatomical prior information that plays a significant role in reducing the number of false positives through disambiguation of collocated lymph nodes and other structures of similar intensity (e.g., esophagus, azygos vein). Presently, only 4 anatomical regions have been used in prior works [8, 12] to distinguish mediastinal LNs from other adjacent structures. We are the first to segment mediastinal nodes by leveraging the anatomical priors of 28 different structures in the body, and thereby account for the aforementioned challenges in the radiology workflow.

In this paper, we present an approach to segment mediastinal LNs in CT studies of the body. Figure 1 shows an overview of the pipeline. We used the LN labels for 89 CT volumes from the public NIH CT Lymph Node dataset, and combined them with the labels for 28 distinct structures in the body obtained through the public TotalSegmentator tool. Three off-the-shelf nnUNet segmentation models were trained end-to-end with this data, and evaluated on a test dataset comprising of 15 patients from an external institution. Our results indicated a performance improvement (measured through Dice scores and Hausdorff distances) over the current state-of-the-art method evaluated on the same test dataset.

## Methods

### Patient sample

We used datasets from two distinct institutions for the purposes of training and testing the 3D nnUNet models. The public NIH CT Lymph Node dataset [7, 21] was used for training, and it comprised of a total of 176 contrast-enhanced CT series from 176 patients. 90 CT volumes were obtained at the level of the chest (mediastinum). Segmentation masks were provided for 388 nodes with a short axis diameter (SAD) $\ge$ 1 cm, which are considered clinically enlarged and abnormal. We accounted for the variability in the SAD measurements by radiologists in this work, and LNs with a SAD $\ge$ 8 mm were considered clinically significant and suspicious for metastasis [3, 19, 20, 22]. The remaining 86 CT volumes were acquired at the abdomen with 595 abdominal LNs annotated. To our knowledge, no underlying disease causes or demographics were provided for the patients in the NIH dataset, and LNs that were smaller than 1 cm were left unannotated.

However upon visual inspection, only 89 of the 90 mediastinal CT volumes had a field-of-view centered around the thorax. Additionally, Bouget et al. [12] provided the ground truth annotations for all the mediastinal LNs in these 89 volumes. In particular, the authors adopted a “conservative” annotation approach and segmented all suspicious regions as lymph nodes. Nodes with any short-axis measurement including those nodes smaller than the suggested RECIST criterion for malignancy of 1 cm were also annotated. All the annotations for the 89 volumes were used to train the models in this work.

The external test dataset (publicly available) from St. Olavs Hospital in Trondheim, Norway [9, 23] comprised of 15 patients with confirmed lung cancer diagnosis. A total of 384 lymph nodes were annotated in this dataset with 143 nodes having a SAD $\ge$ 8 mm and 241 nodes with SAD < 8 mm. As previously mentioned, LNs with a SAD $\ge$ 8 mm were considered clinically significant to account for any variability in radiologist measurements. To our knowledge, this is the largest publicly available dataset in which all mediastinal LNs have been segmented. The dimensions of the contrast-enhanced CT volumes in the test set ranged from (487 $\sim$ 512) $\times$ (441 $\sim$ 512) $\times$ (241 $\sim$ 829) voxels. The volumes in this dataset also contained ”burned-in” metadata (arrows, measurements, descriptions), which can adversarially affect models not trained on such data.

### Anatomical priors

Inspired by prior literature [6, 8, 9, 11–13] on LN segmentation with anatomical priors, we utilized the public TotalSegmentator [24] that was designed to segment over 117 distinct classes in CT volumes. The tool is of tremendous use for various applications, such as personalized risk assessment through body composition analysis [25, 26]. TotalSegmentator was developed using a training set of 1,204 CT exams and encompasses a diverse array of scanners, institutions, and protocols to ensure its versatility and robustness in different clinical settings. The segmentation labels generated by this tool for 28 different structures (e.g., trachea, pulmonary artery etc.) in the body were utilized. After combining them with the lymph node labels, a total of 29 distinct classes were used for training the segmentation models. Incorporation of anatomical priors helped to disambiguate anatomical regions of the body that are of similar intensity as the LNs, such as the heart and esophagus. Furthermore, the primary goal was to segment LNs and not map their stations. Therefore, at test time, the predicted LN labels were retained and the remaining 28 classes were discarded. A complete list of the labels provided by TotalSegmentator is detailed in Supplementary Material Table 3.

### 3D nnUNet

The self-configuring nnUNet segmentation framework [27] was employed to train different configurations for the task of LN segmentation in CT. The nnUNet model is currently the *de-facto* standard for segmentation, and it can be adapted for various datasets and modalities, including CT and MRI. The framework automatically determined the optimal hyper-parameters for training a segmentation model and learned to segment target structures of interest. In this work, 3D low-resolution, 3D full-resolution, and 3D cascade nnUNet configurations were trained and their performances compared.

During training, each configuration of the 3D nnUNet took as input the CT (unwindowed) volume and the corresponding ground-truth masks for 29 different structures. Five-fold cross-validation with different initializations of trainable model parameters for each fold was done. The default nnUNet training scheme was used for all folds and each fold was trained for 1000 epochs. Distinct subsets of training and validation data from the 89 CT volumes were automatically created for each fold. The model learned to segment the target structures of interest in the volume, and iteratively refined it via a loss function. The loss function used by the model was an equally weighted combination of binary cross-entropy and soft Dice losses. This loss function computed a segmentation error that measured the overlap between the prediction and ground-truth. It was optimized using the Stochastic Gradient Descent (SGD) optimizer with an initial learning rate of $10^{-2}$ and a batch size of 1. At test time, the 3D nnUNet predicted the segmentation masks for the structures in the held-out test CT volumes. The remaining classes were discarded at test time. The best model with the lowest loss from each of the 5 folds was used for inference on the test CT volume, and predictions from these five folds were ensembled together.

**Table 1:** 3D Detection results of clinically relevant lymph nodes (SAD $\ge$ 8 mm)

#	Experiment	Anatomy prior	GT	TP	FP	FN	Precision	Sensitivity	F1-score
1	Bouget et al. [12]	Yes	–	–	–	–	–	46.4	–
2	3D nnUNet (Full Res)	No	143	85	5	58	**94**.**4**	59.4	72.9
3	3D nnUNet (Full Res)	Yes	143	84	6	59	93.3	58.7	72.1
4	3D nnUNet (Low Res)	Yes	143	84	5	59	**94**.**4**	58.7	72.4
5	3D nnUNet (Cascade + Low Res)	Yes	143	90	9	53	90.9	62.9	74.4
6	3D nnUNet (Cascade + Full Res)	Yes	143	91	8	52	91.9	**63**.**6**	**75**.**2**

Bold font indicates best results. “–” stands for unreported results

**Table 2:** 3D Segmentation results for clinically relevant and all lymph nodes

#	Experiment	Anatomy prior	LN $\ge$ 8 mm		All LN	
			Dice $\uparrow$	HD $\downarrow$	Dice $\uparrow$	HD $\downarrow$
1	Bouget et al. [12]	Yes	–	–	44.8 ± 13.5	–
2	3D nnUNet (Full Res)	No	62.1 ± 26.6	33.5 ± 21.2	56.2 ± 23.5	44.5 ± 32.2
3	3D nnUNet (Full Res)	Yes	62.5 ± 26.2	25.7 ± 25.2	55.9 ± 23.4	44.3 ± 31.8
4	3D nnUNet (Low Res)	Yes	57.3 ± 27.6	39.2 ± 24.5	48.8 ± 23.2	56.1 ± 32.9
5	3D nnUNet (Cascade + Low Res)	Yes	65.9 ± 23.8	26.9 ± 23.9	57.2 ± 20.7	49.9 ± 30.2
6	3D nnUNet (Cascade + Full Res)	Yes	**67**.**9** ± **23**.**4**	**22**.**8** ± **20**.**2**	**58**.**7** ± **21**.**3**	**41**.**9** ± **32**.**9**

Bold font indicates best results. “–” stands for unreported results

## Experiments

The 3D nnUNet models in our work were trained with the data acquired at the NIH and tested on an external dataset obtained at St. Olavs Hospital (out-of-training distribution). First, the primary experiment was the comparison against the slab-wise UNet designed by Bouget et al. [12], which was evaluated on the same test dataset. Next, the proposed approach with anatomical priors (“fullRes”) was compared against a segmentation model trained without any anatomy priors (“fullRes_noPrior”). Finally, we also determined the capabilities of the following nnUNet configurations with anatomical priors: 3D low-resolution nnUNet (“lowRes”), 3D cascade nnUNet with first-stage predictions from the low-resolution nnUNet (“cascLR”) and from the full-resolution nnUNet (“cascFR”). All experiments were run on a desktop running Ubuntu 20.04 LTS with a NVIDIA V100 GPU with 32GB RAM.

*Metrics* Precision, sensitivity, and F1-score was used to measure the detection performance. The Dice score coefficient (DSC) and symmetric Hausdorff Distance (HD) were used to quantify the segmentation performance. The implementation of these assessment criteria was obtained from the official Medical Segmentation Decathlon challenge [28]. Unlike [9, 12, 13] where post-processing steps were applied to the predicted segmentations, such as removal of nodes through connected component analysis, no post-processing was applied to the predictions from our segmentation models. LNs were only partitioned based on their SAD, and results were computed for all LNs and clinically relevant nodes (SAD $\ge$ 8 mm). The LabelShapeStatisticsImageFilter function [29] in the SimpleITK python package was utilized to compute the Feret diameter and stratify nodes by their SAD (length perpendicular to Feret diameter).

**Fig. 2:**
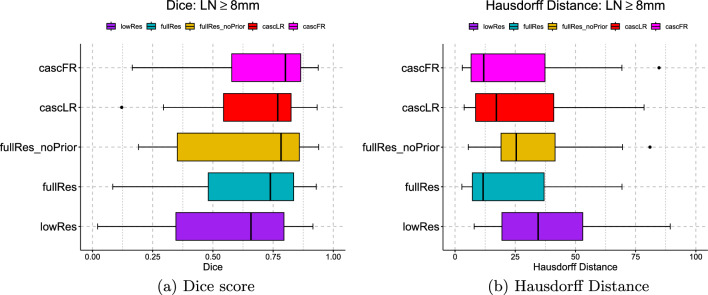
Box plots of the different 3D nnUNet model configurations for the segmentation of mediastinal lymph nodes in the St Olavs dataset. Dice scores and Hausdorff distances are shown for clinically relevant lymph nodes with short axis diameters $\ge$ 8 mm

**Fig. 3:**
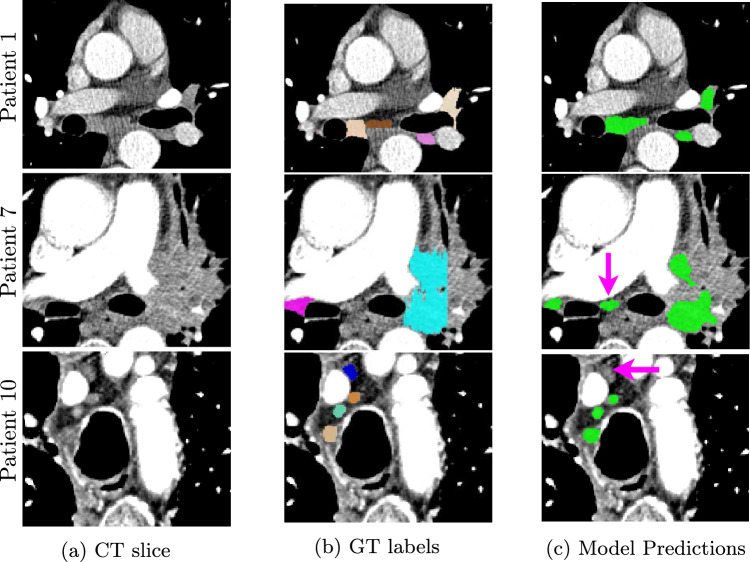
Visual example results of mediastinal LN detection and segmentation in CT. Left column: A slice of the original CT volume for a patient, Middle column: ground truth labels annotated by a radiologist, Right column: Prediction from the 3D cascade nnUNet model. The different colors in the GT correspond to the different stations of the LNs, but for evaluation purposes, they were all considered to belong to one class based on their short axis diameter. For Patient 1 in (c), all four lymph nodes were correctly segmented by the model including the two co-located nodes. For Patient 7 in (c), the model partially captured the large metastatic node (cyan), while it also identified an unmarked node (magenta arrow) in the volume. The unmarked node was considered a false positive for metric computation when it should actually be a true positive. Finally, for Patient 10 in (c), the model missed the lymph node in blue (indicated by magenta arrow)

## Results

Tables 1 and 2 summarize the detection and segmentation performances of the different methods respectively. Box plots in Fig. 2 show the distribution of the Dice scores and Hausdorff distances respectively across the different approaches. Figure 3 provides example outputs from the best performing model.

First, the detection and segmentation results indicated that all the nnUNet configurations (with and without anatomical priors) outperformed the approach proposed by Bouget et al. [12] by $\ge$10%. Next, we compared the 3D full-resolution nnUNet “fullRes” trained with anatomical priors against the same 3D full-resolution nnUNet “fullRes_noPrior” trained without anatomical priors. The “fullRes_noPrior” model had a marginally higher (>1%) detection performance (rows 2 and 3 in Table 1), while the “fullRes” model showed a marginally higher (>1%) segmentation performance (rows 2 and 3 in Table 2). However, the Hausdorff distance error for the 3D “fullRes” model trained with priors decreased by $\ge$4%, which indicated a closer agreement with the ground truth. One potential reason for this was that the model penalized the LN predictions if it encroached on adjacent anatomy that was not the target lymph nodes. In the absence of anatomical priors, this penalty was removed, which consequently increased the Hausdorff distance error. Thus, the presence of anatomical priors was important for LN segmentation.

Then, we compared the performance of the different nnUNet configurations. The 3D “lowRes” nnUNet with anatomy priors achieved similar detection performance as the 3D “fullRes_noPrior” nnUNet. But, the “lowRes” nnUNet fared the worst amongst all configurations in terms of segmentation with the lowest Dice scores and Hausdorff distance errors. In contrast, the 3D cascade nnUNet models attained the best detection and segmentation scores. In particular, the 3D “cascFR” nnUNet with first-stage predictions from the full-resolution nnUNet demonstrated the best LN detection performance for 2/3 metrics (sensitivity and F1-score) with an acceptable level of precision. It also exhibited the highest Dice score and lowest Hausdorff distance error for all LNs and clinically relevant LNs with SAD $\ge$8 mm.

Figure 2 shows the median values of the Dice scores steadily increase and the Hausdorff distances decrease for LNs $\ge$ 8 mm when transitioning from the “lowRes” nnUNet to the cascade “cascFR” nnUNet model. Of note, the 3D “fullRes_noPrior” model without anatomical priors displayed the widest spread of Dice scores and Hausdorff distance errors. This stands in contrast to the distributions of the “fullRes” and “cascFR” nnUNet models, which showed a smaller spread for Dice scores and lower Hausdorff distance errors. The findings signaled the benefit of providing anatomical priors to these models. Again, the 3D “cascFR” nnUNet trained with anatomical priors attained the best median Dice score and lowest median Hausdorff distance.

With respect to the computation time, the 3D “fullRes_noPrior” model took $\sim$1.5 days to complete training with 89 volumes, whereas the “fullRes” model trained with anatomical priors took $\sim$3 days. On the other hand, the 3D “cascFR” nnUNet took $\sim$5 days to complete. For inference, the 3D full-resolution nnUNet (with and without anatomy priors) took an average of $\sim$1.5 min per volume to produce the segmentation labels, while the 3D cascade nnUNet took a little longer, $\sim$3 min per volume.

The publicly available dataset of 15 patients [9, 23] is the only external test dataset currently available with all suspicious LNs entirely annotated. Due to the low number of testing cases (n = 15), a non-parametric Wilcoxon signed-rank statistical test did not yield statistically different results. But, given the clear improvements in the segmentation capabilities, we believe that the addition of more data would provide clearer insights into any performance differences. Nevertheless, these findings again point to the utility of anatomical priors for improved segmentation performance.

## Discussion and conclusion

In this work, we trained various configurations of a 3D nnUNet to segment lymph nodes in mediastinal CT volumes with anatomical priors. As evidenced by prior works [6, 8, 11–13], the utilization of 28 anatomical priors improved the 3D nnUNet’s segmentation of lymph nodes as they provided guidance to the model during training. Despite the provision of a dataset containing fully annotated lymph nodes, the model was penalized if it over-segmented any nodes that encroached into adjacent anatomical regions. The decreased Hausdorff distance errors provide evidence of this effect. Particularly, the 3D full-resolution nnUNet trained without the anatomy priors exhibited higher Hausdorff distance errors. The 3D cascade nnUNet obtained the highest Dice scores and lowest distance errors for all LNs and those with SAD $\ge$ 8 mm, which were considered clinically significant.

A comparison of the proposed nnUNet configurations against recent transformer-based segmentation methods, such as nnFormer [30] or foundation models [31] was not conducted. As noted in prior work [30], the average difference in Dice scores between nnUNet and transformer-based approaches for multi-structure segmentation was <1%. Due to the comparable performance differences, we used only nnUNet models in this work. Foundation models currently support only 2D inputs, whereas the nnUNet models take 3D volumes as inputs. Moreover, the network architecture of foundation models cannot be automatically self-configured for identification of the best training hyper-parameters for a specific dataset. But, the nnUNet framework can be automatically self-configured to provide the best hyper-parameters for training segmentation models, and thus this framework is still the *de-facto* standard for medical segmentation tasks.

Presently, it is impossible for an automated approach to obtain voxel-perfect segmentations of LNs due to technical challenges in the CT acquisition process. The timing and uptake of contrast material can fluctuate, resulting in adjacent regions (e.g., azygos vein) to be similar intensity as the LNs that straddle the mediastinum as seen in Figs. 3(a) and 3(b), which can obscure their shape and size. Despite complete annotations of LNs, it is possible that certain nodes can be missed by a model as shown in Fig. 3(c). Additionally, the manual annotations done by trained radiologists for LNs in CT may not always be complete. For example, in Fig. 3(b), a lymph node was not annotated in the ground truth, but the nnUNet model correctly segmented this missed lymph node. Incomplete ground truth could also reduce the segmentation Dice scores as the correctly detected LN would be incorrectly considered as a false positive instead of a true positive.

Furthermore, the true metastatic nature of a node can only be determined through an invasive biopsy procedure for diagnosis. But, this may not be clinically feasible due to small sizes or anatomic locations. Thus, reliance on CT, PET/CT, or ultrasound imaging markers are few of the non-invasive ways to assess malignancy [12]. Utilizing PET/CT can provide complementary information on metastatic nodes based on their metabolic activity; higher SUV values (regardless of the nodal size) are suspicious for metastatic disease. However, PET/CT is not the initial diagnostic test and is generally performed after CTs first identify a malignancy and areas of metastatic disease; to that end, the initial CT exam must be exhaustively used to derive biomarkers.

One of the main limitations of our work is the inability to disambiguate collocated LNs in the CT volumes due to the diversity of LN shapes and appearances. As pointed out by Bouget et al. [12], this task is often difficult even for an experienced radiologist, and it is expected that the task would be equally, if not more, challenging for an automated method as well. Additionally, we do not tackle the problem of station mapping in this work. Furthermore, the test dataset that we used in this work is relatively small with only 15 patients. Due to clear imbalances in the station-level distributions [12], an extensive data collection and annotation process would be required to address both these issues. This would also enable any statistical differences to be extracted across the different nnUNet model configurations.

As localization of lymph nodes and measurement of suspicious nodes are routine tasks that clinicians perform on a day-to-day basis, our end-to-end anatomical prior-guided approach to segmenting lymph nodes would potentially alleviate the cumbersome nature of the measurement task. Since the models were trained with data that was presumably acquired with a variety of imaging scanners and exam protocols, it is fair to note that our 3D cascade model was sensitive to identifying LNs with SAD $\ge$ 8 mm while maintaining a high detection precision. It holds promise as a tool to report automated measurements, differentiate metastatic from non-metastatic nodes, and flag any concerning LNs that were missed by the reading radiologist.

In summary, the segmentation of mediastinal lymph nodes in CT was explored in our work through the use of anatomical priors. In addition to the LN labels for 89 volumes from the public NIH CT Lymph Node dataset, 28 different structures were also used to train different configurations of off-the-shelf 3D nnUNet models in an end-to-end manner. As post-processing steps were unnecessary, the 3D cascade model was able to achieve the highest segmentation Dice score of 67.9 ± 23.4 and lowest Hausdorff distance errors of 22.8 ± 20.2 for clinically significant LNs with SAD $\ge$ 8 mm. Our results show an improvement of 10% over the current state-of-the-art method that was evaluated on the same test dataset. Mining additional LNs in unannotated CT exams would enable the segmentation performance to be improved over time. Our approach has the potential for improved patient outcomes through the identification of enlarged nodes in initial staging CT exams, while also determining the best options for next steps, whether that be diagnostic biopsy or therapeutic treatment.

### Supplementary Information

Below is the link to the electronic supplementary material.

Supplementary file 1 (pdf 119 KB)
