# Supplementary material for: Segmentation of mediastinal lymph nodes in CT with anatomical priors
Source: Int J Comput Assist Radiol Surg. 2024 May 13;19(8):1537–44. doi: 10.1007/s11548-024-03165-4 (PMC11329534; doi:10.1007/s11548-024-03165-4)
Supplement: Supplementary file 1 — (pdf 119 KB) [file 11548_2024_3165_MOESM1_ESM.pdf]

6 Supplementary Material

Table 3: Complete list of all organs and structures used for training the 3D nnUNet models in this work. 28 classes were generated by TotalSegmentator when it was executed on the 89 CT volumes in the NIH CT Lymph Node dataset. These were combined with the lymph node labels from the NIH CT Lymph Node dataset to yield the final 29 classes for training.

#	Structure	Class ID	Extracted From
1	Body Region Mask	1	TotalSegmentator
2	Lymph Nodes	1	NIH CT LN data (annotated by St Olavs)
3	Spleen	3	TotalSegmentator
4	Kidneys (left & right)	4	TotalSegmentator
5	Gall Bladder	5	TotalSegmentator
6	Liver	6	TotalSegmentator
7	Stomach	7	TotalSegmentator
8	Aorta	8	TotalSegmentator
9	Inferior Vena Cava	9	TotalSegmentator
10	Portal and Splenic Vein	10	TotalSegmentator
11	Pancreas	11	TotalSegmentator
12	Adrenal Glands (left & right)	12	TotalSegmentator
13	Lung (all lobes)	13	TotalSegmentator
14	Skeleton (vertebrae & ribs & pelvis & sacrum & humerus & scapula & clavicula & femur & hip)	14	TotalSegmentator
15	Esophagus	15	TotalSegmentator
16	Trachea	16	TotalSegmentator
17	Heart (Mycardium & Atria & Ventricles)	17	TotalSegmentator
18	Pulmonary Artery	18	TotalSegmentator
19	Iliac Artery	19	TotalSegmentator
20	Iliac Vein	20	TotalSegmentator
21	Small Bowel	21	TotalSegmentator
22	Duodenum	22	TotalSegmentator
23	Colon	23	TotalSegmentator
24	Glutes Max	24	TotalSegmentator
25	Glutes Min	25	TotalSegmentator
26	Glutes Medius	26	TotalSegmentator
27	Autochthon	27	TotalSegmentator
28	Iliopsoas	28	TotalSegmentator
29	Urinary Bladder	29	TotalSegmentator
